# LPP3 mediates self-generation of chemotactic LPA gradients by melanoma cells

**DOI:** 10.1242/jcs.207514

**Published:** 2017-10-15

**Authors:** Olivia Susanto, Yvette W. H. Koh, Nick Morrice, Sergey Tumanov, Peter A. Thomason, Matthew Nielson, Luke Tweedy, Andrew J. Muinonen-Martin, Jurre J. Kamphorst, Gillian M. Mackay, Robert H. Insall

**Affiliations:** 1 Beatson Institute for Cancer Research, Garscube Estate, Switchback Road, Bearsden, Glasgow G61 1BD, UK; 2 Institute of Cancer Sciences, University of Glasgow, Garscube Estate, Switchback Road, Bearsden, Glasgow G61 1BD, UK; 3 York Teaching Hospital NHS Foundation Trust, The York Hospital, York YO31 8HE, UK; 4 Leeds Cancer Centre – Melanoma Institute, Bexley Wing, St James's University Hospital, Beckett Street, Leeds LS9 7TF, UK

**Keywords:** Melanoma, Metastasis, LPA, LPP3, Self-generated gradients, Chemotaxis

## Abstract

Melanoma cells steer out of tumours using self-generated lysophosphatidic acid (LPA) gradients. The cells break down LPA, which is present at high levels around the tumours, creating a dynamic gradient that is low in the tumour and high outside. They then migrate up this gradient, creating a complex and evolving outward chemotactic stimulus. Here, we introduce a new assay for self-generated chemotaxis, and show that raising LPA levels causes a delay in migration rather than loss of chemotactic efficiency. Knockdown of the lipid phosphatase LPP3 – but not of its homologues LPP1 or LPP2 – diminishes the cell's ability to break down LPA. This is specific for chemotactically active LPAs, such as the 18:1 and 20:4 species. Inhibition of autotaxin-mediated LPA production does not diminish outward chemotaxis, but loss of LPP3-mediated LPA breakdown blocks it. Similarly, in both 2D and 3D invasion assays, knockdown of LPP3 diminishes the ability of melanoma cells to invade. Our results demonstrate that LPP3 is the key enzyme in the breakdown of LPA by melanoma cells, and confirm the importance of attractant breakdown in LPA-mediated cell steering.

This article has an associated First Person interview with the first author of the paper.

## Abstract (precis)

**Highlighted Article:** Melanoma cells can create and follow their own gradients of attractant, via a new mechanism by which tumour cells may undergo metastasis.

## INTRODUCTION

Malignant melanoma is the most aggressive form of skin cancer, accounting for the majority of skin cancer-related deaths (American Cancer Society, 2014). Melanoma is highly treatable if diagnosed in its early stages while still localized; however, patient prognosis worsens significantly once metastasis has occurred. Melanoma is often classified according to its localization and growth characteristics, transitioning from early ‘radial growth phase’ (RGP) plaques localized in the epidermis, to proliferative ‘vertical growth phase’ (VGP) tumours that can break through the basement membrane into the dermis, increasing the likelihood of metastasis (Bennett, 2008; Elder, 2015). The factors involved in bringing about melanoma metastasis are still not completely understood. Changes in cell–cell adhesion due to alterations in cadherin expression, and other changes in the gene expression programme related to epithelial–mesenchymal transition (EMT), may play a part by altering the cytoskeletal, invasive and migratory properties of cells and weakening cell–cell junctions (Li et al., 2015). However, in the case of melanoma, other drivers of metastasis are thought to be particularly important. One such driver is chemotaxis, in which cells migrate up gradients of extracellular signals. However, the signals driving melanoma cell migration out of the primary tumour are poorly characterized. One signal that may contribute to melanoma metastasis is the phospholipid signalling molecule lysophosphatidic acid (LPA), which was recently discovered to be a strong chemoattractant for melanoma cells (Muinonen-Martin et al., 2014).

LPA has been implicated in a wide range of signalling pathways and cellular processes including proliferation, migration, survival and adhesion. It consists of a phosphate head group, glycerol backbone and acyl chain of varied length and saturation, characteristics that control biological activity (Jalink et al., 1995). Longer acyl chains and, in particular, unsaturated C:C bonds within the acyl group correlate with greater activity; short acyl chains and complete saturation make the LPA almost inactive (Jalink et al., 1995). The 18:1, 18:2 and 20:4 species (18 or 20 carbons in the acyl chain and one, two or four unsaturated bonds) are thought to be the most active *in vivo*. LPA is found both intracellularly and extracellularly in tissues, and is also a major component of serum (Eichholtz et al., 1993; Baker et al., 2001; Yung et al., 2014). Extracellular LPA may be generated from lysophosphatidylcholine (LPC) via autotaxin, or converted from phosphatidic acid by membrane-bound phospholipase A1. However, source–sink gradients, in which one cell type produces LPA and another removes it to set up a chemotactic gradient, have not been found.

LPA mediates its effects through a family of seven transmembrane G-protein-coupled receptors. Six LPA receptors have been identified (LPAR1–LPAR6), which bind LPA with varying affinity and interact with different combinations of the four Gα proteins: Gα_12/13_, Gα_q/11_, Gα_i/o_ or Gα_s_ (Pasternack et al., 2008; Yung et al., 2014). LPA is broken down by a family of lipid phosphate phosphatases (LPPs). The LPPs are transmembrane enzymes located at the plasma membrane with an extracellular active site, and also on intracellular membranes. They dephosphorylate LPA into biologically inactive monoacylglycerol (MAG). There are three known mammalian LPPs (LPP1–LPP3; also known as PPAP2A, PPAP2C and PPAP2B, respectively) and an additional splice variant of LPP1 (Samadi et al., 2011).

LPA degradation by melanoma cells is of particular interest, as they appear to be able to form gradients with a low LPA concentration within the tumour and high LPA outside it (Muinonen-Martin et al., 2014), thus setting up a ‘self-generated’ gradient directing cells outwards from the primary tumour ([Bibr JCS207514C33][Bibr JCS207514C34]). Although LPA is a well-known inducer of cell motility and the role of LPA signalling in ovarian cancer is well studied (Fang et al., 2002; Tanyi et al., 2003a), it has only recently been shown to be a uniquely powerful chemoattractant for melanoma cells (Muinonen-Martin et al., 2014). Thus LPA gradients could facilitate the exit and metastasis of melanoma cells from the tumour. Here, we investigated the mechanism by which melanoma cells break down LPA, demonstrating that LPP3 is crucial for the formation of self-generated LPA gradients and subsequent chemotaxis and invasion.

## RESULTS

### Melanoma cells respond least efficiently to high concentrations of LPA

LPA is found in serum at varying concentrations of 0.1–10 μM (Eichholtz et al., 1993; Xu et al., 1998; Baker et al., 2001; Fang et al., 2002; Mazzocca et al., 2011; Yung et al., 2014), with an average of ∼1 μM, although this has been shown to increase under pathological conditions including various cancers. In contrast, the *K*_d_ of LPAR1, the most important LPA receptor, is ∼10 nM (Choi et al., 2010). The concentration of LPA in serum – or even the 10% serum used in culture medium – is therefore saturating to most sensitive cells. As a significant difference in receptor occupancy across cells is required for directed chemotaxis, we investigated how melanoma cells are able to sense and respond to these high concentrations of LPA.

To test the response of melanoma cells to increasing concentrations of LPA, we used Insall chambers (Muinonen-Martin et al., 2013, 2014) to visualize chemotaxis, in particular measuring evolution of chemotactic efficiency over time. The use of direct-viewing chambers allowed us to quantify chemotaxis in detail while cells migrated. One straightforward output is the chemotactic index, described by the cosine of the angle between the gradient and the cells' migration (cos θ); positive chemotactic indices indicate migration up-gradient, with a value of >0.1 indicating significant chemotaxis.

**Fig. 1.**
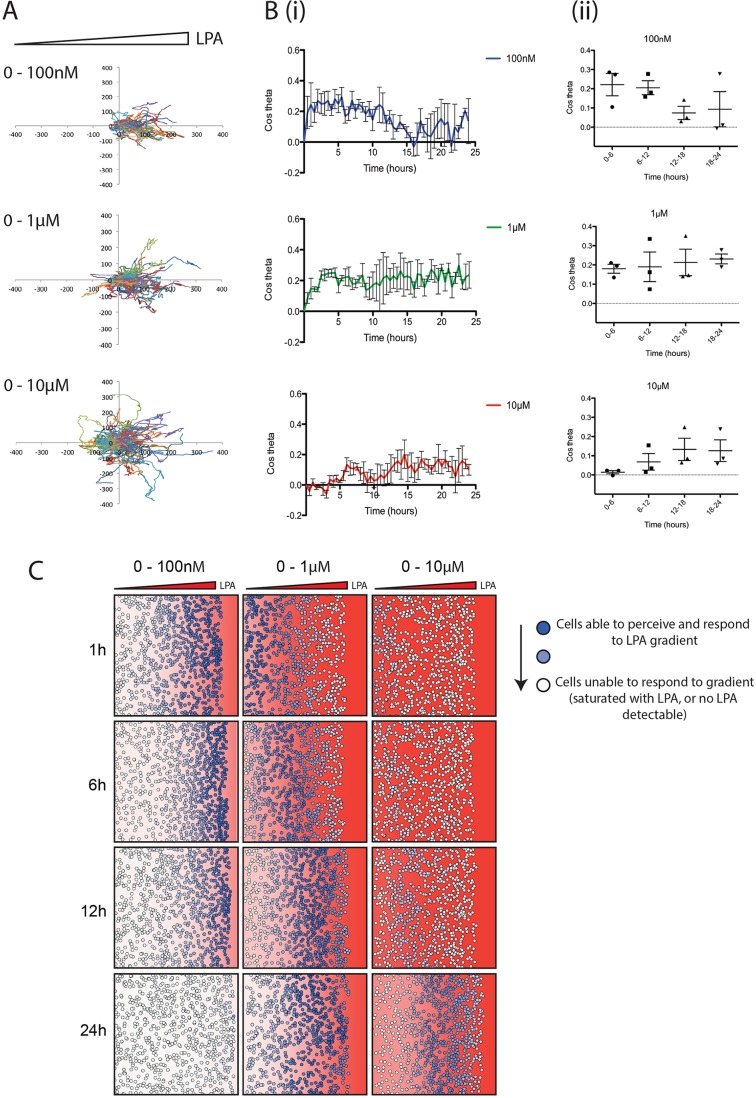
**Higher LPA concentrations delay melanoma cell chemotaxis.** (A) Representative spider plots showing cell tracks of WM239A metastatic melanoma cells moving from serum-free media (on the left) towards 100 nM, 1 μM or 10 μM 18:1 LPA (on the right) in an Insall chemotaxis chamber. Axis scale is in micrometres. (B) Chemotactic response of WM239A cells over time, in the presence of increasing LPA concentration gradients. The mean (±s.e.m.) chemotactic index of cells is shown (i) over 24 h and (ii) across 6 h time increments (*n*>100 cells for each condition, three independent experiments). (C) Representative snapshots over time from a mathematical model simulating cells locally degrading and responding to different concentrations of a freely diffusing chemoattractant (with similar kinetics and receptor affinities to LPA/LPAR). The red background represents the LPA concentration within the gradient and corresponding level of LPAR saturation. Solid red indicates a concentration of 1 μM LPA or higher, and thus high receptor saturation. The colour fades linearly to white, which indicates a concentration of 0 μM LPA, and thus no receptor saturation. Circles in dark blue represent cells able to perceive the LPA gradient, while circles fading to white represent cells increasingly unable to perceive the LPA gradient, either because no LPA is present or because too much LPA is present, oversaturating their receptors.

When imposing an external chemotactic gradient across the cells, we found that increasing concentrations of LPA did not increase the efficiency of directional migration of WM239A metastatic melanoma cells (Fig. 1). Although the WM239A cells were able to migrate towards LPA at all concentrations between 100 nM and 10 μM, (Fig. 1A), the efficiency of chemotaxis evolved differently as the concentration rose. With an initial concentration of 100 nM LPA, cells were immediately chemotactic (Fig. 1Bi), with a mean chemotactic index of 0.22 (±0.06 s.e.m.) (Fig. 1Bii). This dropped over time, most likely reflecting the degradation of LPA. When using 1 μM LPA, the initial level of chemotaxis was slightly lower (0.18±0.2) (Fig. 1Bi), but increased to 0.23±0.3 by 18–24 h. In contrast, at high concentrations of LPA (10 μM), chemotaxis was initially negligible, increasing over 24 h. Cells never reached the same chemotactic index as at lower concentrations of LPA. These results support the hypothesis that cells' receptors are saturated at high concentrations of LPA, making it impossible for cells to perceive local changes, but they eventually break down LPA to sub-saturating levels that allow them to sense a gradient.

It is not currently technically possible to measure dynamic spatial LPA gradients directly. We therefore used a mathematical model that accurately represents the way cells modify gradients while responding to them (Tweedy et al., 2016a) to further assess melanoma cell responses to varying levels of LPA. Figure 1C shows such a simulation of cells in different LPA gradients. At low initial LPA concentrations (0–100 nM), most cells at the front respond at first as they are experiencing the steepest resolvable LPA gradient, but behind this front there is very little LPA present for cells to respond to. Over time, the cells at the front also stop chemotaxing as the LPA is locally degraded. At intermediate concentrations (0–1 μM), cells at the front are totally saturated and few initially respond. However, chemotaxis increases as the LPA is degraded to a level that cells can respond to. At the highest concentrations (0–10 μM), chemotaxis only starts once the majority of LPA is broken down, which takes at least 12 h; even after this time, receptor saturation limits the ability of cells to perceive a gradient. Measurement of the chemotactic index of these simulated cells over time (Fig. S1) demonstrates the close agreement between model and measured data. This supports our hypothesis that changes in chemotaxis over time are mediated by the melanoma cells breaking down LPA as they respond to it.

### LPP3 is required for LPA breakdown by melanoma cells

**Fig. 2.**
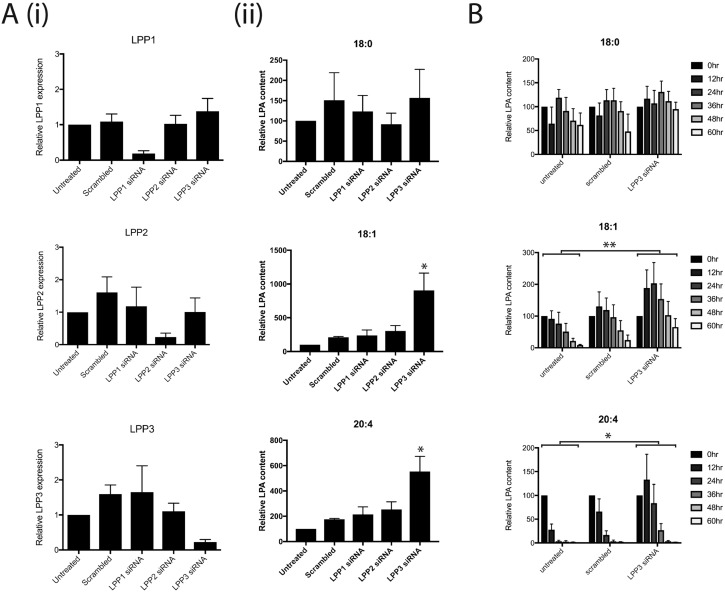
**LPP3 is required for LPA breakdown by melanoma cells.** (A) siRNA knockdown of the LPA breakdown enzymes LPP1, LPP2, LPP3 or a scrambled control, in WM239A melanoma cells; (i) representative qPCR confirming knockdown of gene expression; (ii) mass spectrometry quantifying relative LPA content in media cultured for 24 h with WM239A cells. Different forms of LPA are shown: (top to bottom) 18:0, 18:1 and 20:4 (*n*=4, *P≤0.05 for LPP3 versus all other conditions, one-way ANOVA). (B) Time course of LPA breakdown by WM239A cells either untreated or treated with control siRNA or siRNA targeting LPP3 alone (*n*=4, *P≤0.05; **P<0.01 for untreated versus PPAP2B, two-way ANOVA comparing the area under the curve).

Having demonstrated that the saturating LPA found in serum must be broken down in order to stimulate melanoma cell chemotaxis, we determined which enzymes from melanoma cells degrade LPA. We used siRNA to knock down expression of genes encoding each relevant mammalian LPP: LPP1, LPP2 and LPP3. Robust and specific knockdown was obtained in WM239A melanoma cells (Fig. 2Ai). Next, LPA-containing medium was incubated for 24 h with WM239A cells, which were untreated or transfected with either a scrambled non-targeting siRNA or siRNAs targeting one LPP gene. The medium was analysed by mass spectrometry for LPA content, with the synthetic 17:0 form added during processing as an internal control. There was no significant difference between the different conditions in degradation of the less biologically active 18:0 form of LPA (Fig. 2Aii). However, LPP3 knockdown resulted in significantly higher levels of 18:1 and 20:4 LPAs (Fig. 2Aii), which are the biologically active forms responsible for signalling (Jalink et al., 1995). Over 60 h, we found a clear delay in the rate of 18:1 and 20:4 LPA degradation when LPP3 was knocked down (Fig. 2B).

A slight initial increase in 18:1 and 20:4 LPA at earlier time points suggests there is a background level of LPA production that is eventually normally compensated for by LPP3-mediated LPA breakdown. The addition of the autotaxin inhibitor HA130 leads to decreased LPA levels after 24 h (Fig. S2), confirming that autotaxin can mediate further production of LPA over the saturating initial level.

Together, these results demonstrate that LPP3 is the key enzyme for degradation of the biologically active forms of LPA in melanoma cells.

### An assay to determine the role of LPA breakdown in self-generated chemotaxis

**Fig. 3.**
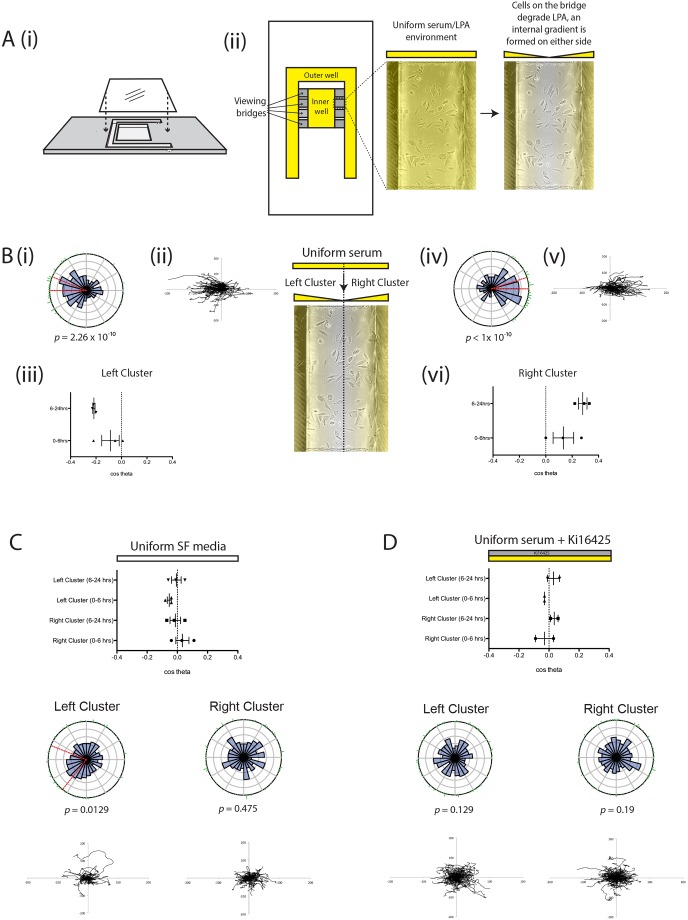
**Spread assay to assess LPA breakdown and chemotaxis.** (A) (i) Side view of an Insall chemotaxis chamber showing inner and outer wells and positioning of the coverslip with cells adhered to it (adapted from Susanto et al., 2016); (ii) bird's eye view of an Insall chamber, indicating the position of the viewing bridges (in yellow). (B) A uniform serum environment produces differential directional movement of the cells on the left (left cluster; i–iii) and right (right cluster; iv–vi) of the bridge, as quantified by mean (±s.e.m.) chemotactic index (iii, vi) or shown in representative rose plots with Rayleigh tests for directionality (i, iv) and the corresponding spider plots (ii, v) (*n*>100 cells per experiment, three independent experiments). In conditions where there is a uniform serum-free (SF) environment (C) or uniform serum with the addition of 10 μM Ki16425 (D) there is no significant difference in cell directionality between the left and right clusters (*n*>100 cells per experiment, two to three independent experiments).

To investigate self-generated chemotaxis of melanoma cells towards LPA, we adapted the Insall chamber chemotaxis assay (Muinonen-Martin et al., 2013) (Fig. 3Ai). Rather than imposing a gradient by adding serum-free and serum-containing medium to opposite wells, we added serum-containing medium to both, to create an initially uniform environment. As the viewing bridge is only 20 µm high, the local volume of liquid is restricted, and the absolute amount of LPA is very small. However, the wells are far deeper, and thus contain a larger reservoir of LPA, which is consequently depleted much more slowly. Thus local degradation of LPA by cells on the bridge would be expected to create a double outward-facing gradient, where cells would be attracted to LPA diffusing from the nearest well (Fig. 3Aii). As predicted, in this assay cells initially positioned on the left side of the bridge (designated the ‘left cluster’) generally moved further to the left (Fig. 3Bi–iii) and cells on the right (‘right cluster’) moved rightwards (Fig. 3Biv–vi, Movie 1). This effect was significant for both clusters (Rayleigh test for directionality; *P*<10^−9^) (Fig. 3Bi,iv). Furthermore, directionality of cells in both clusters increased over time, from a slightly positive (rightwards) initial value to a substantial chemotactic index after 6 h for the right cluster (Fig. 3Bvi), and corresponding negative (leftwards) values for the left cluster (Fig. 3Biii). We hypothesized that this delay was due to the time taken to break down LPA and create a gradient.

To confirm that this effect was specific to LPA, and not a random effect reflecting the proximity of cells to the edge of the bridge, we tested the movement of cells in uniform serum-free conditions (Fig. 3C, Movie 2) and in a uniform serum-containing environment containing the LPA receptor antagonist Ki16425 (Fig. 3D, Movie 3). In both cases, we observed no significant directional movement of cells in either cluster.

### LPP3 knockdown inhibits LPA breakdown-mediated chemotaxis

**Fig. 4.**
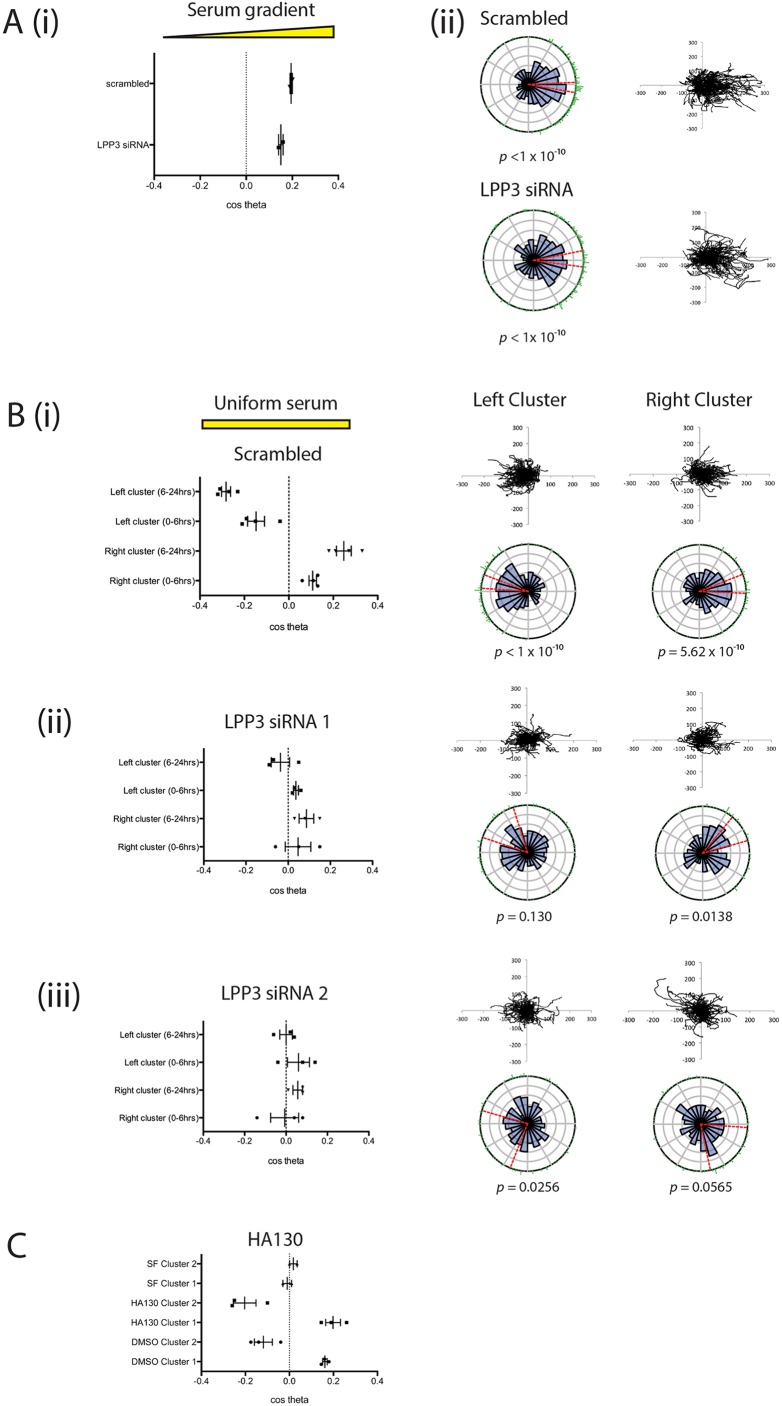
**LPP3 knockdown inhibits chemotaxis in uniform serum.** (A) LPP3 knockdown (LPP3 siRNA 1) does not inhibit chemotaxis of melanoma cells in the presence of a serum gradient, as assessed by (i) chemotactic index or (ii) rose plot and spider plot (*n*>100 cells per experiment, two independent experiments). (B) LPP3 knockdown inhibits differential chemotaxis of melanoma cells in a uniform serum gradient, as shown when comparing (i) scrambled siRNA controls against cells targeted with two different siRNAs against LPP3 (ii, iii) (*n*>100 cells per experiment, four independent experiments; *P*<0.05 comparing clusters 1 and 2 of scrambled versus LPP3 siRNA 1 and 2, one-way ANOVA). (C) Chemotaxis of melanoma cells in a uniform serum gradient in the presence of either DMSO or 500 nM HA130 to inhibit autotaxin function, or in a uniform serum-free (SF) gradient (*n*>100 cells per experiment, three independent experiments).

This assay was used to demonstrate that inhibiting LPA breakdown by LPP3 knockdown prevents the formation of outward-facing gradients, and thus prevents self-generated chemotaxis. We used siRNA to transiently knock down LPP3 expression, using two individual oligos that consistently reduced LPP3 expression relative to scrambled controls (Fig. S3). Firstly we demonstrated that LPP3 knockdown itself did not affect the response of melanoma cells to LPA in an external serum gradient (Fig. 4A). LPP3 siRNA-treated cells and scrambled-siRNA controls had similar mean chemotactic indices (0.195 and 0.15, respectively; Fig. 4Ai), and both populations showed highly directed migration towards serum with Rayleigh *P*-values of <10^−10^ (Fig. 4Aii). However, in the self-generated chemotaxis assay, LPP3 knockdown cells displayed obviously different behaviour from controls. Control cells produced gradients that triggered chemotaxis of left and right clusters in opposite directions (Fig. 4Bi). In contrast, LPP3 knockdown caused a significant reduction in chemotaxis of both clusters, with virtually no directional migration (*P*<0.05; Fig. 4Bii, Movie 4). This was confirmed using the second independent siRNA targeting LPP3 (*P*<0.01; Fig. 4Biii).

If LPA production via autotaxin was important in cell steering, we would expect the opposite results to what we observe. Wherever cell density was high, we would expect to see more LPA and therefore an attractive stimulus, so cells would tend to aggregate rather than dispersing outwards. However, as autotaxin is widely described as a promoter of metastasis, we directly tested its effects. We performed the assay for cell dispersal in the presence of the autotaxin inhibitor HA130 (Fig. 4C, Movie 5). Inhibition of autotaxin did not inhibit differential chemotaxis of melanoma cells in this assay; if anything the inhibition of background LPA production enhanced chemotaxis in response to self-generated gradients. This demonstrates that degradation, rather than production of the chemoattractant LPA, is the key process required for production of self-generated gradients and subsequent cell migration.

### LPP3, LPA and 3D invasion

**Fig. 5.**
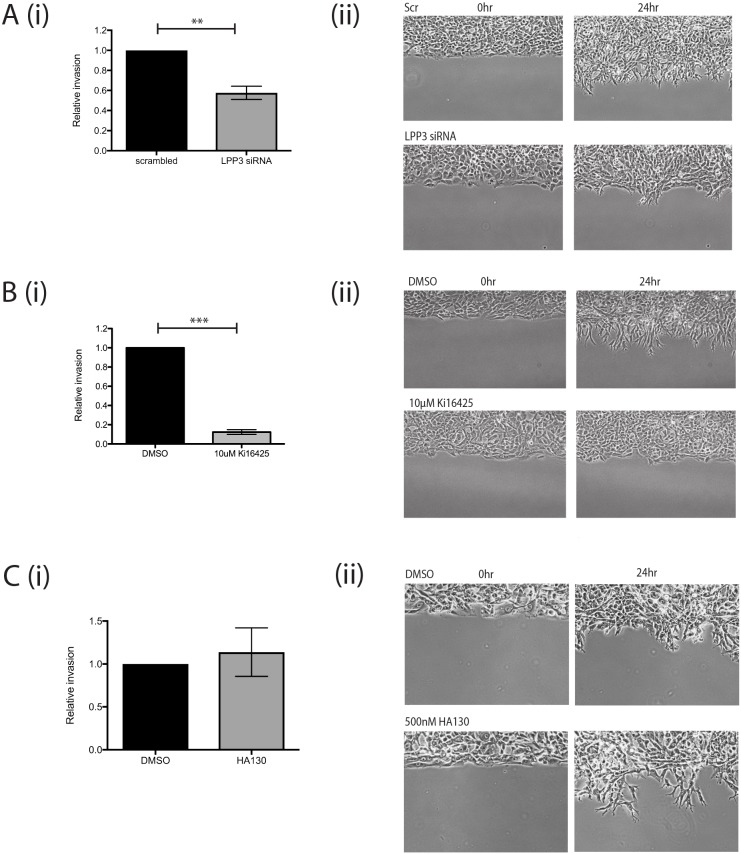
**LPP3 knockdown inhibits melanoma cell invasion in 2D.** (A) (i) Quantification of invasion of WM1158 metastatic melanoma cells transfected with either a scrambled control siRNA or siRNA targeting LPP3 (LPP3 siRNA 1) in a CIA; mean+s.e.m. shown (*n*=4 independent experiments, **P<0.01, Student's *t*-test); (ii) representative images of cell invasion at 0 and 24 h. (B) (i) Quantification of invasion of WM1158 cells treated with DMSO control or 10 μM Ki16425 in a CIA (*n*=3 independent experiments, ***P<0.0001, Student's *t*-test); (ii) representative images of cell invasion at 0 and 24 h. (C) (i) Circular invasion assay using WM1158 cells treated with DMSO control or 500 nM HA130 (*n*=3 independent experiments); (ii) representative images of cell invasion at 0 and 24 h.

We also investigated if melanoma cell invasion involved self-generated gradients, and whether LPP3 was required to generate them. First we used circular invasion assays (CIAs). The CIA enables direct visualization of cell migration in a manner similar to direct-view chambers, but in a setting that mimics invasion into 3D extracellular matrix, by forcing the cells to invade Matrigel (Yu and Machesky, 2012). We used a different metastatic melanoma cell line, WM1158, as it displayed higher baseline levels of invasion. We observed that LPP3 knockdown resulted in reduced levels of invasion compared with controls (*P*<0.01; Fig. 5A; Movie 6). As described earlier, this implies that outward-facing gradients of LPA are more important than the LPA concentration itself, and that LPP3 is instrumental in forming them.

LPP3 has been reported to be important for cell–cell adhesion and extracellular matrix modulation by endothelial cells (Humtsoe et al., 2010). To verify that the reduction in melanoma cell invasion we measured was due to LPA gradient formation being blocked and not due to other LPP3-mediated interactions, we tested whether blocking LPA signalling specifically would inhibit invasion as well. Treatment of cells before and during the assay with Ki16425 produced a similar effect, markedly inhibiting invasion compared with controls (*P*<0.0001; Fig. 5B; Movie 7). In contrast, addition of the autotaxin inhibitor had no effect on melanoma cell invasion into the matrix (Fig. 5C; Movie 8). Thus, the formation of self-generated LPA gradients by LPP3 appears to be a key factor in the chemotaxis and invasion of melanoma cells.

**Fig. 6.**
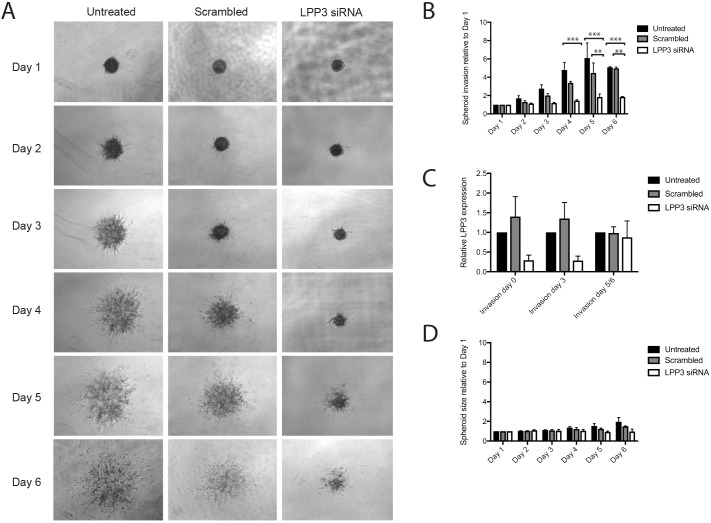
**LPP3 knockdown inhibits melanoma cell invasion in 3D.** 3D spheroid invasion assay using WM852 metastatic melanoma cells either left untreated or transfected with either a scrambled control siRNA or LPP3 siRNA 1. (A) Representative images of spheroid invasion over 6 days following the addition of invasion matrix. (B) Quantification of 3D invasion relative to spheroid size on day 1. Means+s.e.m. are shown (*n*=4 independent experiments, **P<0.01; ***P<0.001, two-way ANOVA). (C) qPCR of *LPP3* expression in WM852 at days 0, 3 and 5/6 following addition of invasion matrix (*n*=3 independent experiments). (D) Quantification of spheroid size relative to spheroids on day 1, in the absence of any invasion matrix (*n*=3 independent experiments).

Finally, we also examined spheroids to determine if LPP3 expression and self-generated gradients were important for invasion in a 3D assay. We used the metastatic melanoma cell line WM852, which was the most clearly invasive line available in spheroid assays, treated with siRNAs targeting LPP3 or scrambled controls. Spheroids were imaged for 6 days following the addition of the invasion matrix, with the control spheroids producing many invasive protrusions outwards from the centre and displaying eventual dispersal of the cells from the central mass (Fig. 6A). In comparison, the LPP3 knockdown spheroids displayed reduced invasion (Fig. 6B). Although LPP3 siRNA knockdown was only transient and the effect dissipated by day 6 (Fig. 6C), the effect was still potent enough to shorten and reduce the number of protrusions being generated, in addition to blocking cell dispersal outwards from the spheroid. Differences in proliferation appeared to have a minimal effect, as in the absence of invasion matrix the control spheroids were only slightly larger than the LPP3 knockdown spheroids (Fig. 6D).

Overall, these results show that LPA is important in multiple assays for melanoma cell migration and invasion, and LPP3 plays a key part in these responses by shaping externally provided LPA into chemotactic gradients that steer individual cells away from any area with a high density of cells.

## DISCUSSION

We have previously demonstrated the efficiency of self-generated gradients as a driver of cell migration (Tweedy et al., 2016a) and that self-generated LPA gradients are key factors in melanoma cell chemotaxis and thus metastasis (Muinonen-Martin et al., 2014). Here, we have delineated the mechanism by which melanoma cells may be triggered to migrate away from the tumour via self-generated gradients.

LPA is ubiquitous in serum, and also found in healthy tissues. Serum LPA is often present at a high base concentration of 1 μM (and significantly higher in some cancers). At this concentration, receptors at the front and rear of cells are all saturated, so cells are unable to detect LPA gradients and trigger chemotaxis. In order to read the gradient, cells need time to degrade the LPA to a level that does not saturate the LPA receptors, so that they can perceive spatial variations in LPA concentration as local differences in receptor occupancy. The higher the LPA concentration, the longer before the cells can respond. Similarly, the more cells available to degrade the LPA, the better they are able to respond (Muinonen-Martin et al., 2014). Our data demonstrate that LPP3 is the key enzyme required for melanoma LPA breakdown. Furthermore, we have shown using various different metastatic melanoma cell lines in a range of 2D and 3D assays that LPP3 is essential for the production of a self-generated LPA gradient that can stimulate melanoma cell chemotaxis and invasion.

### LPP3 plays a non-redundant role in LPA breakdown

Although all three LPPs are thought to have similar catalytic activity, they appear to have non-redundant functions, as evidenced by the different phenotypes observed in *Ppap2a* (LPP1), *Ppap2b* (LPP3) and *Ppap2c* (LPP2) knockout mice. While *Ppap2a* and *Ppap2c* knockout mice display only very mild phenotypes (Zhang et al., 2000; Tomsig et al., 2009), *Ppap2b* (LPP3) knockouts are embryonic lethal due to abnormal development of the vasculature (Escalante-Alcalde et al., 2003). This may indicate the need for self-generated LPA gradients in development; elsewhere, self-generated S1P gradients have been shown to be essential for T-cell function in the lymph system (Thangada et al., 2010). We also found that knockdown of LPP1 and LPP2 did not have any overt effect on LPA degradation, while LPP3 knockdown resulted in initially increased levels of LPA and slower rates of breakdown over time. Thus, LPP3 appears to be the dominant lipid phosphatase required for LPA breakdown by melanoma cells.

### LPP3 is responsible for melanoma-mediated self-generated gradients

As there are no reagents currently available to directly visualize LPA degradation, we have developed a robust assay that can be used to examine and quantify cell responses to the formation of localized self-generated gradients. We have shown that after ∼6 h, melanoma cells in uniform serum-containing medium migrate towards the nearest LPA source. This effect is lost in serum-free medium, indicating that it is not merely an effect of random migration. Most importantly, LPP3 knockdown also abolished this effect, establishing the link between LPA breakdown and the ability of melanoma cells to form gradients in uniform serum. Although LPP3 is also capable of breaking down the bioactive lipid S1P, it is unlikely that S1P is involved in these self-generated gradients as the specific LPA receptor antagonist Ki16425 fully blocked chemotaxis.

There is substantial literature implicating the phosphodiesterase autotaxin, which generates LPA from LPC, in cancer spread and chemotaxis (Nam et al., 2000; Saunders et al., 2008). However, self-generated chemoattractant gradients generated by autotaxin would be expected to be high where cells were most dense (at the centre of tumours), and therefore oppose cancer cell spreading and invasion. We have shown that in contrast to LPP3 knockdown, the addition of the autotaxin inhibitor HA130 does not abolish the divergent chemotaxis of melanoma cells in our assay; in fact the effect is slightly enhanced. This may be due to the elimination of a background level of LPA production, which could blur the effect of the self-generated gradient. Thus, these data confirm that it is the breakdown of LPA and formation of self-generated gradients that is the key factor responsible for driving melanoma cell migration, rather than autotaxin-mediated LPA production. As LPA has a split role – as well as being a chemoattractant, it is an exceptionally potent mitogen – it seems more likely that autotaxin's principal role is to promote tumour growth. Breakdown of attractants, like the LPP3-mediated process we have described, generates gradients that lead away from the tumour and is a more likely driver of metastasis.

### A role for LPP3 in melanoma metastasis

Although chemotaxis assays are useful for determining cellular responses to chemoattractants in 2D (Insall and Andrew, 2007), they do not emulate the tissue environment faced by tumour cells when metastasizing. Here, we have used CIAs and 3D spheroid invasion assays to examine melanoma cells invading a matrix-filled environment. Although the serum-containing medium in a CIA/spheroid assay is not flow-restricted as in an Insall chamber, the LPA may be broken down locally within the Matrigel component containing the cells, enabling self-generated LPA gradients to be maintained. Indeed, we found that LPP3 knockdown significantly inhibited LPA-dependent melanoma cell invasion, reinforcing the role of self-generated LPA gradients in facilitating melanoma metastasis. Thus far, we have not found evidence of LPP3 having significantly altered expression in metastatic melanoma cell lines compared with non-metastatic melanoma cells. Rather, we hypothesize that upon tumourigenesis and proliferation of the melanoma cells, the localized increase in melanoma cell numbers (relating directly to the tumour thickness, known to pathologists as the ‘Breslow thickness’ and a key diagnostic) is sufficient to induce the LPP3-mediated degradation of surrounding LPA above background levels.

These findings present an interesting potential target for anti-metastatic therapy when treating melanoma. Interestingly, LPP1 and LPP3 have been targeted in potential therapies for ovarian cancer ([Bibr JCS207514C30][Bibr JCS207514C31]; Benesch et al., 2016). However, these therapies aimed to increase LPP expression in order to reduce overall LPA levels, thereby diminishing its pro-tumourigenic effects. In contrast, we would hypothesize that increasing LPP3 expression in melanoma cells would speed up their ability to create self-generated gradients, and thus accelerate migration out of the tumour. Thus, to diminish metastasis, LPP3 could potentially be neutralized to inhibit LPA breakdown around a tumour site.

In future, it will be interesting to test the universality of self-generated gradients in other processes and systems. LPP3 has previously been shown to be essential in depleting S1P within the thymus and spleen, setting up S1P gradients that stimulate lymphocyte migration, while its homologue has been shown to be involved in germ cell migration in *Drosophila* (Renault et al., 2004; Breart et al., 2011; Ramon-Perez et al., 2015). Thus, signalling lipid breakdown to form self-generated gradients may be common in both normal physiology and metastasis of various types of cancer, warranting further investigation into this simple yet powerful mechanism of cell steering.

## MATERIALS AND METHODS

### Cell lines and reagents

WM239A, WM1158 and WM852 melanoma cell lines were obtained from the Wellcome Trust Functional Genomics Cell Bank (Biomedical Sciences Research Centre, St George's, University of London, UK). Cells were cultured in RPMI medium (Invitrogen) supplemented with 2 mM l-glutamine (Gibco) and 10% fetal bovine serum (FBS; PAA Laboratories) and confirmed mycoplasma free.

1-Oleoyl-2-hydroxy-*sn*-glycero-3-phosphate (18:1) and 1-arachidonoyl-2-hydroxy-*sn*-glycero-3-phosphate (20:4) LPA was obtained from Avanti. FlexiTube GeneSolution sets of four siRNAs (Qiagen) were used to target LPP1, LPP2 and LPP3. LPP3 siRNA 1 was oligo 6 from the ON-TARGET plus siRNA range (J-017312-06-0005; GE Dharmacon; siRNA sequence: GGGACUGUCUCGCGUAUCA), LPP3 siRNA 2 was oligo 7 from the FlexiTube siRNA range (SI03043761, Qiagen; siRNA sequence: AGCGATCGTCCCGGAGAGCAA), scrambled non-targeting siRNA control was AllStars negative control siRNA (Qiagen). Ki16425 (Cambridge Bioscience) was used at a final concentration of 10 μM. HA130 (Echelon Biosciences) was used at a final concentration of 500 nM.

### Insall chamber chemotaxis assay

Cells were plated onto fibronectin-coated glass coverslips at a density of 7×10^4^ cells/ml and then serum-starved overnight in serum-free RPMI medium prior to use as previously described (Muinonen-Martin et al., 2013; Susanto et al., 2016). Serum-free (SF) medium consisted of RPMI with 2 mM l-glutamine, penicillin/streptomycin (Invitrogen) and 0.5% BSA (Sigma), while serum-containing medium contained an additional 10% FBS. LPA-containing medium consisted of SF medium with LPA at the indicated final concentrations.

Insall chambers were imaged on a Nikon TE2000-E inverted time lapse microscope equipped with MetaMorph (Molecular Devices), a motorized stage (Prior Scientific, Cambridge, UK) and Perfect Focus System enclosed in a plexiglass box humidified and heated to 37°C with 5% CO_2_. Each viewing bridge on the Insall chamber was imaged with a 10× objective every 30 min over 24 h.

Cells were manually tracked using the MTrackJ plugin for ImageJ (http://rsb.info.nih.gov/ij). All cells on the bridge at the beginning of the experiment were tracked, excluding cells that died, to the end of the experiment or until the cells moved off the viewing bridge. Chemotactic index was calculated as described (Muinonen-Martin et al., 2014).

### siRNA knockdown

Cells were transfected with scrambled or gene-targeting siRNA using Lullaby transfection reagent (Oz Biosciences). Cells were transfected twice with 25 nM siRNA, once every 48 h, then assayed 48 h after the second transfection.

To assess gene knockdown, RNA was extracted from cells using an RNeasy mini kit (Qiagen) and cDNA synthesized from 0.5 μg samples using a SuperScript III reverse transcriptase kit (Life Technologies) as specified by the manufacturer. qPCR was performed using PerfeCTa SYBR green FastMix (Quanta), with cDNA analysed in triplicate using a 7500 fast real-time PCR system (Applied Biosciences); data were extracted using Applied Biosystems 7500 software version 2.0. QuantiTect primer kits (Qiagen) were used to assess gene expression of GAPDH, PPAP2A/LPP1, PPAP2B/LPP3 and PPAP2C/LPP2, with all gene expression normalized against GAPDH as an internal control using the comparative Ct method (Schmittgen and Livak, 2008).

### Mass spectrometry

Cells were plated in a 6-well dish at a density of 1×10^5^ per well, then treated with siRNA. Twenty-four hours after the final transfection of siRNA, old medium was removed and 2 ml of fresh LPA or serum-containing RPMI was added to each well. Media used for LPP knockdown experiments after 24 h was serum-free RPMI with 10 µM 18:0, 18:1 and 20:4 LPA added. Media used for LPP3 knockdown time course experiments was RPMI with 10% serum. One millilitre of supernatant was collected at the indicated time points and a standard amount of synthetic 17:0 LPA was added to the sample prior to butanol extraction (Muinonen-Martin et al., 2014). LPA was analysed using a QExactive orbitrap coupled to a Dionex UltiMate 3000 LC system (Thermo Scientific). The LC parameters were as follows: 4 µl of sample was injected onto a 1.7 µm particle 100×2.1 mm ID Waters ACQUITY CSH C18 column, which was kept at 50°C throughout the analysis. A gradient system of (A) aqueous 0.05% ammonium hydroxide and (B) 0.05% ammonium hydroxide/methanol was used, with a linear gradient of 0.3 ml/min from 50 to 90% B over 7 min rising to 100% B within 1 min and maintaned for 2 min. Thereafter, the column was returned to initial conditions and equilibrated for another 4 min. Lysophospholipids were analysed in electrospray negative ionization mode, with spray voltage 3 kV, capillary temperature 300°C, sheath gas flow rate 50, and auxiliary gas flow rate 10. The resolution was set to 70,000, automatic gain control was set to 1×10^6^ with a maximum injection time of 200 ms and the scan range was 300–700 m/z. Peak areas were determined using MAVEN (Melamud et al., 2010; Clasquin et al., 2012) and concentrations were normalized to the LPA (17:0) standard.

### Circular invasion assay

The CIA was carried out as previously described (Yu and Machesky, 2012; Susanto et al., 2016). Briefly, an Ibidi culture insert was adhered to the centre of a 35 mm low μ-dish (Ibidi), then 1×10^6^ WM1158 cells were plated in the dish around the insert. The following day the insert and supernatant medium were removed and 200 μl of Matrigel (Corning) diluted 1:1 with chilled PBS was added and left to set for 1 h at 37°C; 1 ml serum-containing medium was then added to the dish prior to imaging. If treated with DMSO, Ki16425 or HA130, cells were incubated for 15 min in medium containing the inhibitor/vehicle before removing the culture insert. DMSO/Ki16425/HA130 was also added to the Matrigel and medium added prior to imaging. The level of invasion after 24 h was determined using ImageJ and calculated relative to the scrambled/DMSO treated controls.

### Spheroid invasion assay

WM852 cells were treated with siRNA as described above, then harvested 24 h after the second hit of siRNA. The spheroid invasion assay was performed as per instructions using a Cultrex 96-well 3D Spheroid BME Cell Invasion Assay kit (Trevigen). Briefly, cells were resuspended in the Spheroid Formation Extracellular Matrix and 1×10^3^ cells were plated per well (in triplicate minimum per condition). The cells were left to form spheroids for 3 days, then Invasion Matrix was added to the invasion condition wells and culture medium was added to the control wells. Spheroids were imaged each day for 6 days following addition of the invasion matrix, using a brightfield microscope. Relative invasion was analysed using ImageJ by thresholding the spheroid area and measuring the number of pixels within that area. Each measurement per day was divided by the measurement taken on day 1 (following addition of the invasion matrix) and calculated as a percentage of spheroid area on day 1.

### Chemotaxis simulation model

The agent-based model, modified from Tweedy et al. (2016a), simulated cells locally degrading a freely diffusing chemoattractant. Diffusion was simulated explicitly on a grid using a central differences approximation to the diffusion equation. The wells were modelled as large, finite and well-mixed reservoirs in contact with the grid. Cell movement was biased by the receptor occupancy difference across the cell, with receptor occupancy at a point *x* given by θ=c(*x*)/[c(*x*)+*K*_d_].

### Statistics

All graphs (excluding spider and rose plots) and statistics (excluding Rayleigh tests) were generated using GraphPad Prism 6. Spider plots were generated using Microsoft Excel. Rose plots were generated using the ‘circular’ package in R. Circular confidence intervals were denoted in red, and were calculated using the approach outlined by Upton and Fingleton (Upton and Fingleton, 1989). The *P*-values for the circular range test were calculated using the formula stated in Mardia and Jupp (Mardia and Jupp, 1999), and the *P*-values for the Rayleigh test were calculated using the method outlined in Mardia and Jupp (Mardia and Jupp, 1999).

## Supplementary Material

Supplementary information
