# Supplementary material for: LPP3 mediates self-generation of chemotactic LPA gradients by melanoma cells
Source: J Cell Sci. 2017 Oct 15;130(20):3455–66. doi: 10.1242/jcs.207514 (PMC5665449; doi:10.1242/jcs.207514)
Supplement: Supplementary information [file joces-130-207514-s1.pdf]

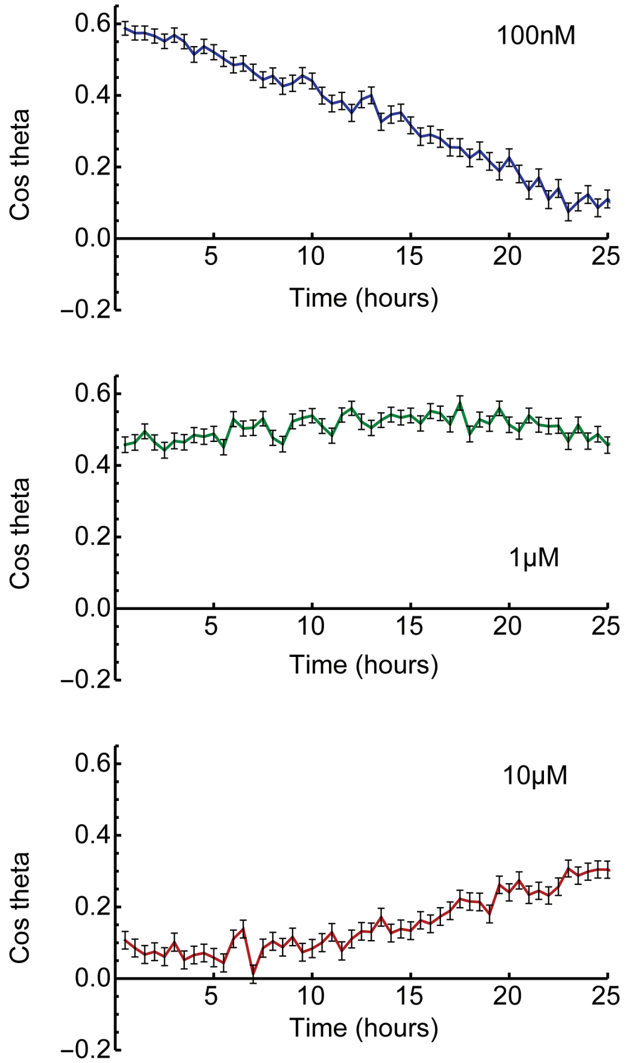

Supplementary Figure 1

Quantification of the cos theta of simulated cells (depicted in Figure 1C) over a 24 hour time period.

Journal of Cell Science • Supplementary information

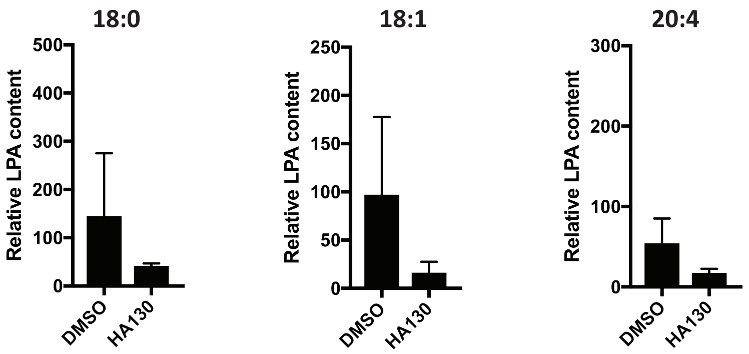

Supplementary Figure 2

Mass spectrometry quantifying relative LPA content. Cells are incubated for 24 hours with serum-containing media in the presence of either DMSO or 500nM HA130 to inhibit autotaxin. Different forms of LPA are shown: (left to right) 18:0, 18:1 and 20:4, showing levels of LPA at 24 hours relative to 0 hours. (n=2 independent experiments). Autotaxin inhibition results in decreased LPA levels, indicating there is a background level of LPA production.

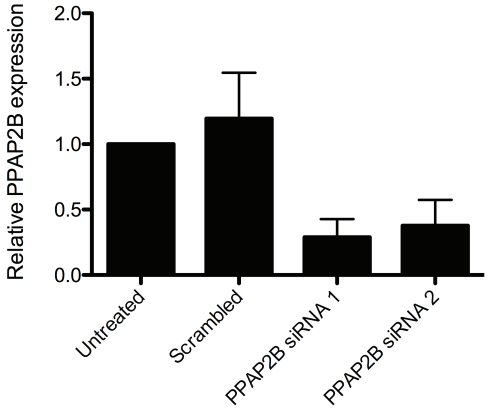

Supplementary Figure 3

Relative expression of PPAP2B in WM239A cells assessed using qPCR. WM239A cells were either left untreated, or treated with siRNA targeting a scrambled control or PPAP2B. RNA was extracted 48 hours after the second siRNA treatment. n= 7, error bars denote SEM.

Journal of Cell Science • Supplementary information

Supplementary Movies

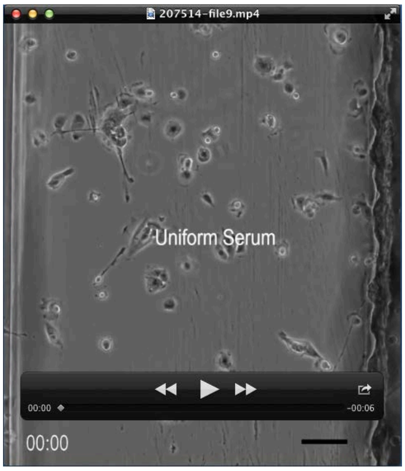

Supplementary movie 1

Representative timelapse movie of WM239A cells in a uniform serum environment. Cells in the "right cluster" display tracks in red and generally move to the right of the bridge over time, while those in the "left cluster" display tracks in yellow and generally move to the left. Scale bar in bottom right corner is 50µm.

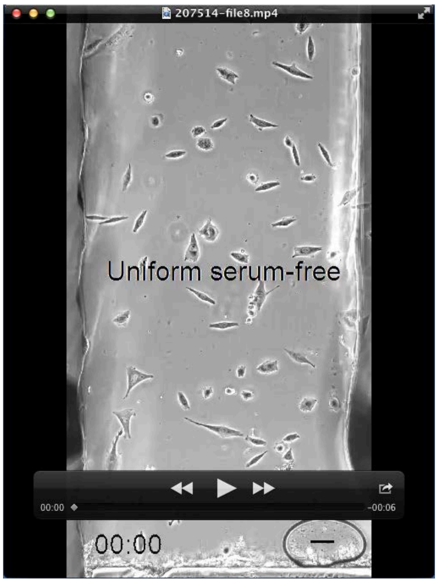

Supplementary movie 2

Representative timelapse movie of WM239A cells in a uniform serum-free environment. Scale bar in bottom right corner is 50µm.

Journal of Cell Science • Supplementary information

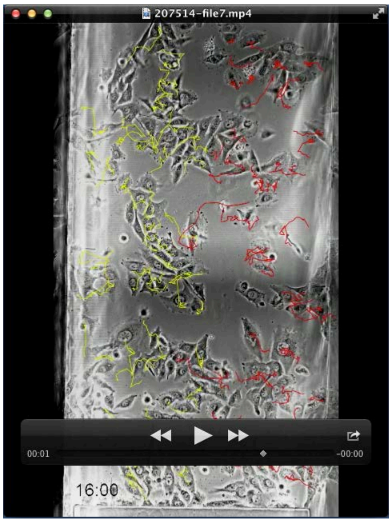

Supplementary movie 3

Representative timelapse movie of WM239A cells in a uniform serum environment in the presence of 10µM Ki16425. Scale bar in bottom right corner is 50µm.

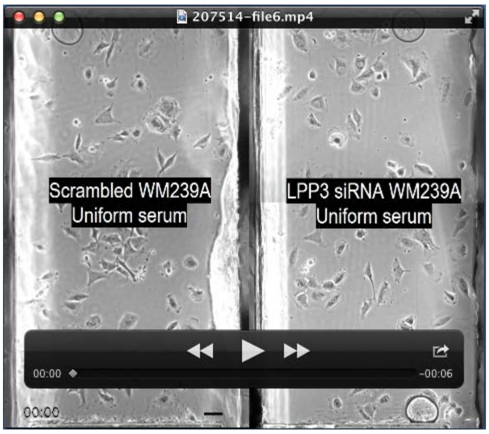

Supplementary movie 4

Representative timelapse movie of WM239A cells, treated with either a scrambled control siRNA or siRNA targeting LPP3, in a uniform serum environment. Scale bar in bottom right corner is 50µm.

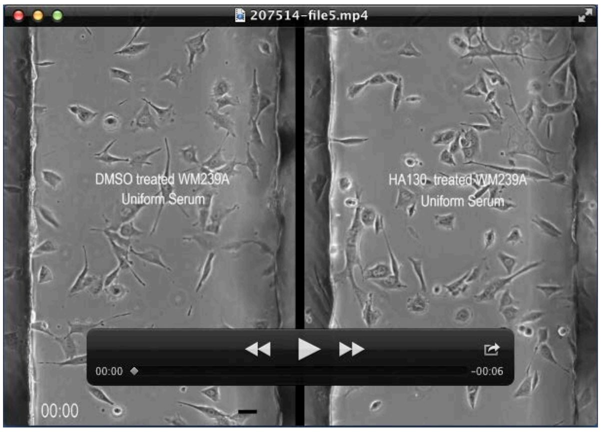

Supplementary movie 5

Representative timelapse movie of WM239A cells, treated with either DMSO as a control or 500nM HA130, in a uniform serum environment. Scale bar in bottom right corner is 50μm.

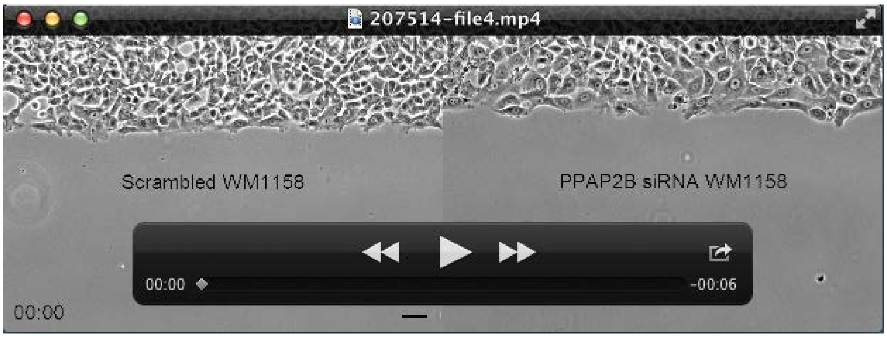

Supplementary movie 6

Representative timelapse movie of WM1158 cells, treated with either a scrambled control siRNA or siRNA targeting LPP3, in a circular invasion assay. Scale bar in bottom right corner is 50μm.

Journal of Cell Science • Supplementary information

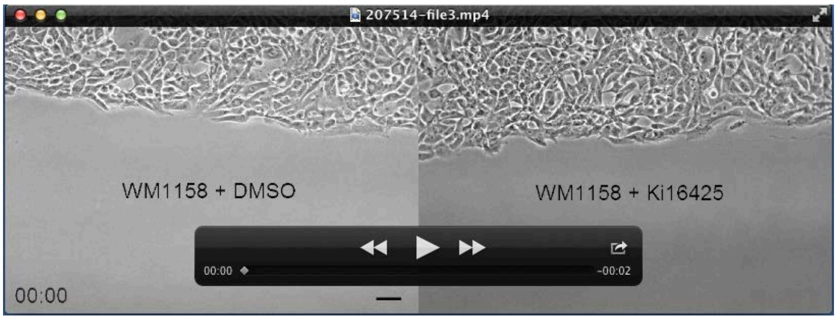

Supplementary movie 7

Representative timelapse movie of WM1158 cells, treated with either a DMSO control or 10µM Ki16425, in a circular invasion assay. Scale bar in bottom right corner is 50µm.

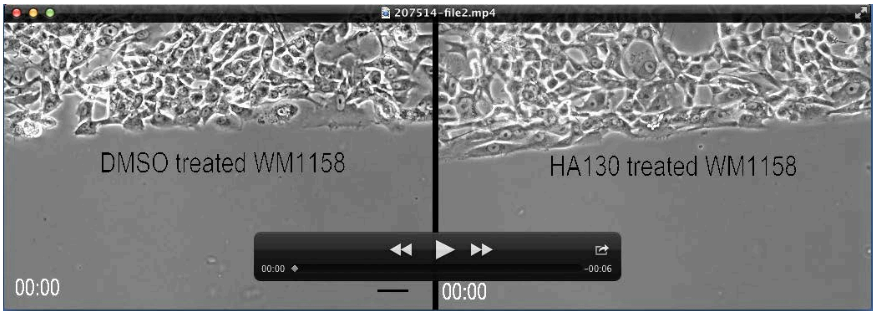

Supplementary movie 8

Representative timelapse movie of WM1158 cells, treated with either a DMSO control or 500nM HA130, in a circular invasion assay. Scale bar in bottom right corner is 50µm.

Journal of Cell Science • Supplementary information
